# Comparison of Changes in Eye Findings of Premenopausal and Postmenopausal Women

**DOI:** 10.7759/cureus.14319

**Published:** 2021-04-06

**Authors:** Ramazan Birgul, Gokce Turan

**Affiliations:** 1 Department of Ophthalmology, Ministry of Health Kirikhan State Hospital, Hatay, TUR; 2 Department of Obstetrics and Gynecology, Gazi University, School of Medicine, Ankara, TUR

**Keywords:** menopause, corneal thickness, refractive value, ocular axial length, blepharitis

## Abstract

Objectives

This study aimed to compare the presence of blepharitis, ocular tension (OT) with corneal thickness, refractive values and ocular axial length changes in premenopausal and postmenopausal women.

Materials and methods

Thyroid-stimulating hormone (TSH), follicle-stimulating hormone (FSH), luteinizing hormone (LH), oestradiol (E2), refractive values with prolactin, refractions in horizontal and vertical meridians, OT values, ocular axial length, central corneal thickness and the presence of blepharitis were evaluated in 153 premenopausal patients and 142 postmenopausal patients.

Results

Statistically significant differences were found for the values for right eye OT (P < 0.001), left eye OT (P < 0.001) and presence of blepharitis. There appears to be no relationship between corneal refractive values corneal thickness or ocular axial length.

Conclusions

Woman in postmenopausal period should be examined by ophthalmologist because of the significant increase in blepharitis and OT.

## Introduction

Menopause is defined as the absence of menstruation in women for 1 or more years [1]. Structural and functional changes occur during the postmenopausal period, including ocular changes [2]. These changes are caused by alterations in hormones, especially oestrogen (E2) deficiency [2,3]. The presence of E2, progesterone and androgen receptors in the lacrimal gland, the meibomian gland, conjunctiva and corneal epithelium form the basis of these changes [4]. The effects of E2 on the cornea, where light is diffracted in the eye, vary. The eye features most affected by these changes are the central corneal thickness and refraction in the horizontal and vertical meridians of the cornea [2,4,5]. Studies have also found that E2 affects ocular tension (OT) [6,7]. The changes in these ocular components can decrease functional effectiveness in terms of visual physiology [5].

Most of the recent studies related to the effects of the postmenopausal period on eye health have focused on eye dryness [4,5,8]. Because of hormonal imbalances during this period, tear production and stability are deteriorated and ocular tissues become vulnerable to microorganisms [9]. This is mainly due to the changes in the meibomian gland as well as the lipid layer that forms the outermost tear layer, which becomes thinner, reducing the quality of tears [10]. Generally, this decrease in the lipid layer occurs due to infection and inflammation of meibomian glands, known as posterior blepharitis [11,12]. Blepharitis is a general condition characterized by infection and inflammation of eyelids, including the roots of eyelashes. Bacteria such as *Staphylococcus epidermidis* and *Propionibacterium acnes* are among the most common agents [13].

This study aimed to compare the refraction, corneal refraction in horizontal and vertical meridians, central corneal thickness, OT, ocular axial length changes and presence of blepharitis in premenopausal and postmenopausal women.

## Materials and methods

This prospective case-control study was conducted between January 2018 and July 2019 in a second-line hospital. Premenopausal and postmenopausal patients admitted to the gynaecology outpatient clinic for routine gynaecological control in the study after receiving informed consent from all participants and approval from the ethics committee (ethics committee approval no: 2019/38). Postmenopausal women were defined as those who had not menstruated for one year or more. Patients with oophorectomy-induced artificial menopause, a history of premature menopause, systemic disease, history of ocular trauma or surgery, inflammatory eye disease, diabetes mellitus (DM), glaucoma, corneal pathology or conjunctival pathology were excluded from this study. Additionally, patients receiving hormone replacement therapy (HRT), who were pregnant or who wore contact lens were also excluded. Patients included in the study were divided into two groups: postmenopausal (study group) and premenopausal (control group). The ages and gravida parity values of the patients were recorded, and gynaecological examinations were performed. Thyroid-stimulating hormone (TSH), follicle-stimulating hormone (FSH), luteinizing hormone (LH), E2 and prolactin levels of all participants were recorded.

After the gynaecological examination, all participants were given an eye examination. Refraction values and keratometric values in the horizontal and vertical meridian (dioptre[D]) of both eyes were measured with an ARK-700 auto-refractometer. OT values (mmHg) were measured with Reichert 7 air tonometry. Ocular axial length (mm) and central corneal thickness (µm) were measured with Topcon noncontact microscopy. The presence of blepharitis was determined with biomicroscopy by observing inflammation of the eyelid skin and eyelash follicles, erythema, squamous debris, shedding of eyelashes, foamy tears and obstruction of the meibomian gland.

The demographic findings, laboratory values and ophthalmologic findings of both groups were recorded and compared with each other. E2 values and eye examination findings were evaluated by correlation analysis.

In this study, statistical analyses were performed using SPSS 20.0 (IBM, Armonk, NY, USA). Descriptive statistics for continuous (quantitative) variables are expressed as means and standard deviations, while categorical variables are expressed as numbers (n) and percentages (%). The suitability of the variables to normal distribution was evaluated by visual and analysis methods, and parametric tests were used due to their suitability for normal distribution. Chi-square and Fisher’s exact tests were used to calculate the demographic characteristics of the patients. Pearson’s rank correlation test was used for univariate correlation analysis. The two groups of dependent and independent data were analyzed with Student’s t-test. The ANOVA test was used when three or more variables were available. A P-value less than 0.05 was considered significant.

## Results

Between January 2018 and July 2019, a total of 322 patients were admitted to the gynaecology outpatient clinic of a second-line hospital. Of these, 15 patients had a history of glaucoma, 5 patients had previous ocular surgery, 4 patients had a history of premature menopause, and 3 patients had a hysterectomy and bilateral oophorectomy. These 27 patients were excluded from the study, and the remaining 295 patients were included in the study. The patients were divided into two groups: 142 premenopausal patients and 153 postmenopausal patients. Demographic data, hormone values and ophthalmologic findings of the patients are listed in Table 1.

**Table 1:** Demographic data, laboratory findings and eye examination findings. FSH: follicle-stimulating hormone; TSH: thyroid-stimulating hormone; LH: luteinizing hormone; E2: oestradiol; OT: ocular tension.

Measurement	Postmenopausal patients (n = 142) (mean ± SD)	Premenopausal patients (n = 153) (mean ± SD)	P-value
Age (years)	42.95 ± 2.15	43.00 ± 2.09	0.620
Gravida	3.35 ± 1.67	3.24 ± 1.81	0.950
Parity	1.28 ± 1.09	1.35 ± 1.42	0.690
FSH	24.10 ± 11.67	11.75 ± 8.60	0.020
LH	16.67 ± 11.82	17.72 ± 6.76	0.820
E2	95.91 ± 104.53	117.77 ± 90.35	0.270
TSH	1.93 ± 1.02	1.77 ± 1.12	0.540
Prolactin	12.94 ± 7.32	12.63 ± 7.59	0.870
Refraction value			
Right eye	0.17 ± 1.39	-0.47 ± 1.11	0.020
Left eye	0.20 ± 1.48	-0.42 ± 0.94	0.020
OT			
Right eye	17.11 ± 1.73	15.71 ± 2.72	<0.001
Left eye	17.26 ± 2.27	16.05 ± 2.44	<0.001
Horizontal keratometry			
Right eye	43.16 ± 1.50	43.21 ± 1.44	0.880
Left eye	43.28 ± 1.75	43.31 ± 1.51	0.930
Vertical keratometry			
Right eye	43.42 ± 1.49	43.95 ± 1.65	0.150
Left eye	43.53 ± 1.60	43.87± 1.53	0.310
Axial measurement value			
Right eye	23.02 ± 0.79	22.90 ± 0.90	0.550
Left eye	22.96 ± 0.87	22.81 ± 0.87	0.440
Pachymetry value			
Right eye	545.45 ± 28.74	537.50 ± 34.28	0.360
Left eye	547.82 ± 28.04	537.68 ± 34.84	0.200
Blepharitis			
Present	99 (69.70%)	81 (52.90%)	<0.001
None	43 (30.29%)	72 (47.00%)	

There were no significant differences between the participants’ age, gravida and parity values. While FSH levels of postmenopausal patients were significantly higher than those of premenopausal patients (P = 0.02), no differences were found between the two groups regarding TSH, LH, E2 and prolactin values. Significant differences were found between the groups regarding right eye refraction (P = 0.02), left eye refraction (P = 0.02), right eye OT (P < 0.001) and left eye OT (P < 0.001) values. There were no significant differences between the values for right eye horizontal keratometry, left eye horizontal keratometry, right eye vertical keratometry, left eye vertical keratometry, right eye axial, left eye axial, right eye central corneal thickness and left eye central corneal thickness. The presence of blepharitis in the postmenopausal group was significantly higher than in the premenopausal group (P < 0.001).When the correlation analysis between the E2 value and eye measurements was examined, no significant difference was observed (Table 2).

**Table 2:** Correlation analysis of the relationship between E2 value and eye measurements. OT: ocular tension.

Measurement	P-value
Refraction value	
Right eye	0.66
Left eye	0.68
OT	
Right eye	0.30
Left eye	0.78
Horizontal keratometry	
Right eye	0.26
Left eye	0.32
Vertical keratometry	
Right eye	0.13
Left eye	0.12
Axial value	
Right eye	0.16
Left eye	0.41
Central corneal thickness	
Right eye	0.67
Left eye	9.52

## Discussion

The perimenopausal period is characterized by the loss of sex hormones and is accompanied by a series of changes in the body. Regarding the visual system, changes can occur to the corneal and ocular surfaces [14]. This study mainly examined the effects of E2 on these two tissues in the postmenopausal period.

The effect of postmenopausal E2 depletion on the lacrimal gland, the meibomian gland, conjunctiva and cornea is different in each tissue. The effect of E2 on the meibomian gland is proinflammatory; its effect on the lacrimal gland and the corneal epithelium is unclear but is believed to be either pro-inflammatory or anti-inflammatory [15]. Studies have reported that E2 changes corneal thickness and that corneal thickness significantly decreases as E2 decreases [4,16]. However, another recent study reported that the relationship between E2 deficiency and reduced corneal thickness was non-significant [17]. In our study, although E2 was decreased in postmenopausal patients, corneal thickness was increased, but the results were not significant. During the postmenopausal period, other hormones besides E2 also decrease in normal physiology. For example, studies have reported that decreased thyroxine hormone, which has receptors in the cornea, is correlated with increased corneal thickness during menopause [18,19]. This suggests that changes in corneal thickness in postmenopausal people are not only related to E2 but other hormones as well.

In a study examining the relationship between E2 and corneal refraction, there was no significant correlation between changes in E2 and corneal refraction in the postmenopausal period; however, a significant correlation was found between horizontal corneal refraction and E2 [20]. In addition, horizontal refraction was slightly increased in that study. In other studies, no significant changes were found in horizontal and vertical corneal refractions in the postmenopausal period [4,20]. In our study, although both vertical and horizontal refraction levels were decreased, this decrease was not significant. The correlation analysis revealed a relationship between vertical refraction and E2, but it was not significant.

Changes in the refraction of the eye primarily result from changes in the cornea, lens and axial length [21]. In our study, we showed that the relationship between decreased corneal refraction and increased corneal thickness and ocular axial length in the postmenopausal period was not significant. The fact that refraction is negative (-) in the premenopausal period and positive (+) in the postmenopausal period was attributed to hypermetropic changes due to the cataract course in the lens as a result of aging rather than hormonal changes.

One study showed that the decrease in E2 in postmenopausal women increases the ocular tension in different ways [7]. It is thought that E2 reduction has an antagonistic effect on the carbonic anhydrase pump in the corneal endothelium, decreasing the uveoscleral flow and increasing the episcleral venous pressure [7,16]. Thus, ocular tension is increased. In accordance with the literature, we also found significantly increased ocular tension in postmenopausal women.

E2 has a proinflammatory effect on meibomian glands, which results in reduced lipid production from the meibomian glands located on the eyelids [15]. This creates two major consequences. First, the lipid layer (the outermost layer of the tear layer) decreases, resulting in eye dryness [22]. Accordingly, most of the recent studies in the literature have focused on the incidence of eye dryness in the postmenopausal period [5,9,23]. Second, the meibomian gland orifices are occluded, which increases the eyelids’ exposure to microorganisms due to the impaired circulation there. Thus, a condition of blepharitis, characterized by inflammation and infection in the eyelids, may develop [9,12]. No studies have directly investigated the incidence of blepharitis in the postmenopausal period, making this study novel. In our study, the blepharitis rate was significantly increased in the postmenopausal period. In normal physiology, the incidence of blepharitis is expected to decrease in the postmenopausal period, which is characterized by a decrease in E2. This paradoxical situation suggests that other hormones have an effect on meibomian glands as well. It is known that androgens have an anti-inflammatory effect on meibomian glands, reducing the incidence of blepharitis [24]. In the postmenopausal period, there is a decrease in androgens along with a decrease in E2 [9]. Thus, the increased incidence of blepharitis in the postmenopausal period is likely due to this decrease in androgens.

## Conclusions

Many hormones, especially E2, cause ocular changes in postmenopausal women. There appears to be no relationship between the decrease in corneal refractive values and increased corneal thickness or ocular axial length. However, the presence of blepharitis and increased OT values are related. Thus woman in postmenopausal period should be examined by ophthalmologist because of significant increase in blepharitis and OT.
